# “Varicose Zone of Alarm” in Pulmonary Tuberculosis

**Published:** 1915-11

**Authors:** 


**"Varicose Zone of Alarm" in Pulmonary Tuberculosis.** Lombardi describes in about 90% of cases, venous varicosities near the spinous processes of the 7th cervical and 1st to 3d dorsal vertebrae. The varicosities may be very small. Slight oedema and pain may accompany them. They indicate apical lesions. The explanation is as follows. It seems to us to require further elucidation. "The spinal-dorcal net has branches that, passing through the transverse apophysis, unite in the neck with the vertebral veins and with the intercostal veins in the chest. The vertebral veins emerge from the transverse apophysis of the sixth cervical vertebra and terminate in the innominate. The intercostal veins of the first two or three spaces of the left side reunite near the vertebral bodies and form the left superior intercostal vein, with the homologous bronchial vein. On the contrary, on the right side, the superior intercostal vein empties into the azygos major."

